# Persistent effectiveness of CGRP antibody therapy in migraine and comorbid medication overuse or medication overuse headache - a retrospective real-world analysis

**DOI:** 10.1186/s10194-024-01813-3

**Published:** 2024-07-04

**Authors:** Armin Scheffler, Jale Basten, Lennart Menzel, Dominik Fiebelkorn, Wolfgang Alexander Becker, Vincent Breunung, Hannah Schenk, Christoph Kleinschnitz, Michael Nsaka, Diana Lindner, Dagny Holle

**Affiliations:** 1 Department of Neurology and Center for Translational Neuro- and Behavioral Sciences (C-TNBS), West German Headache Center, University Hospital Essen, University Duisburg-Essen, Hufelandstr. 55, Essen, 45147 Germany; 2 Department of Medical Informatics, Biometry and Epidemiology, Ruhr University Bochum, Universitätsstraße 105, Bochum, 44789 Germany

**Keywords:** Erenumab, Galcanezumab, Fremanezumab, MOH, Chronic migraine, Detoxification, MO

## Abstract

**Background:**

Management of patients with migraine who have concomitant medication overuse (MO) or medication overuse headache (MOH) is a major problem in clinical practice. Detoxification of acute analgesics before or during initiation of prophylactic therapy has long been recommended although this concept has recently been questioned. Additionally, relapse after detoxification is a common problem. This real-world study analyses the initial and sustained effectiveness of prophylactic migraine therapy with CGRP (receptor) antibodies without prior detoxification in patients with comorbid MO or MOH for up to one year.

**Methods:**

A retrospective real-world analysis was performed on 291 patients (episodic migraine (EM) with MO (EM-MO; *n* = 35), EM without MO (EM-noMO; *n* = 77), chronic migraine (CM) with MOH (CM-MOH; *n* = 109), CM without MOH (CM-noMOH; *n* = 70). All patients began treatment with either erenumab (*n* = 173), fremanezumab (*n* = 70) or galcanezumab (*n* = 48) without prior detoxification. Data were available for up to 12 months of treatment. Responder rates for monthly headache days (MHD), monthly migraine days (MMD) and monthly acute medication intake (AMD) were analysed.

**Results:**

All groups showed a significant reduction in MHD, MMD and AMD at the last observed time point compared to baseline. In patients with CM and MOH, 60.6% (66/109) no longer fulfilled the definition of MO or MOH and a further 13.8% (15/109) had only EM-MO. In the EM cohort, 89% (31/35) of MO patients lost their MO during therapy. MHD and AMD 30% responder rates were comparable for CM-MOH and CM-noMOH (MHD: CM-MOH: 56.0% vs. CM-noMOH: 41.4%, *p* = 0.058, AMD: CM-MOH: 66.1% vs. CM-noMOH: 52.9%, *p* = 0.077). MMD responder rate did not differ significantly (after Bonferroni adjustment) (CM-MOH: 62.4% vs. CM-noMOH: 47.1%, *p* = 0.045, α = 0.017). After successful initiation of therapy, 15.4% of the initial CM-MOH patients relapsed and met the criterion for CM-MOH at the end of follow-up. There were no antibody specific differences in response to therapy.

**Conclusions:**

Our data confirms the effectiveness of CGRP antibody treatment in migraine patients with additional MOH or MO in a real-world setting. Low relapse rates after initial successful therapy support an early start of CGRP antibody treatment in patients with MOH or MO.

**Trial registration:**

No registration, retrospective analysis.

**Graphical Abstract:**

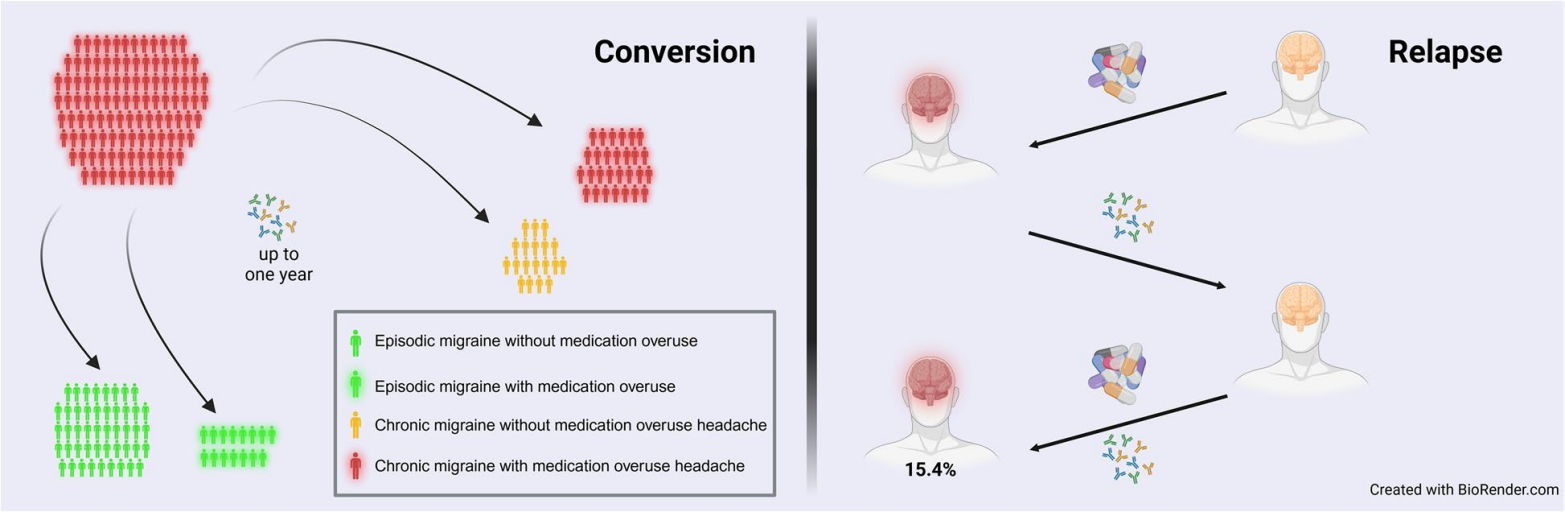

## Introduction

Overuse of acute medication can lead to a chronification of migraine. In general, any type of acute medication overuse (MO) can lead to medication overuse headache (MOH) in the presence of a primary chronic headache disorder. In migraine, triptans and non-opioid analgesics are commonly used as acute medications. To define MO due to triptans, patients must have a monthly acute medication intake (AMD) of triptans for at least 10 days, whereas to define MO due to non-opioid analgesics (e.g. NSAID, excluding triptans), an AMD of at least 15 days is required. This use must continue for at least 3 months. A MOH can be diagnosed when patients with chronic headache disorders (e.g. tension-type headache or chronic migraine (CM)) have MO (see ICHD-3 classification [1]). 

MOH is associated with a major impairment of the patient's quality of life [2] and also causes major health costs [3, 4]. Risk factors for developing a MOH are female gender, psychiatric comorbidities, pre-existing pain and lifestyle-related factors. Although the mechanism for developing a MOH is not clear, direct influences of analgesics and sensitisation of pain fibers, among other factors, have been hypothesised [5].

To date, the best therapy for patients with MOH and MO is still controversial. In the past, it was assumed that the acute medication had to be discontinued before prophylactic therapy can be successful [6]. In the meantime, several randomised clinical trials have been conducted, suggesting that some prophylactic drugs (i.e. topiramate, onabotulinumtoxin A and human monoclonal antibodies targeting calcitonin gene-related peptide or its receptor) are effective even if MO is not discontinued before the start of therapy [7, 8].

Nonetheless, the previous treatment options often do not have a long-lasting effect. Even if the MOH was initially well treated and no longer present, a significant proportion of patients experience a relapse after an initial period of successful therapy, so that these patients once again fulfill the definition of MOH. Numerous studies show that relapse is a relevant problem in MOH treatment [9–12]. So far, there is a lack of data on whether this also applies to patients who are treated with CGRP (receptor) antibodies.

This real-world analysis examines the therapy of CGRP (receptor) antibodies in terms of initial and sustained effectiveness in patients with migraine and MOH or MO.

## Materials and methods

A retrospective analysis of clinical routine data including headache diaries, questionnaires and medical documentations was performed. Data were collected every three months. Monthly headache days (MHD), monthly migraine days (MMD) and AMD were defined as the average value of the previous three months. All patients were treated at the West German Headache Center, Department of Neurology, University Hospital Essen, Germany, between November 2018 and May 2023. The independent ethics committee of the University Hospital Essen (19–9004-BO) approved the analysis. All patients gave written informed consent for potential scientific analysis of their data. Due to the retrospective analysis of internal routine data, a special written informed consent was not required for this specific analysis.

All patients with baseline values and at least one post-treatment time point (after three, six, nine or twelve months) were included. They were classified as having episodic migraine (EM) or chronic migraine (CM) according to the ICHD-3 criteria [1]. Additionally, patients with a high frequency of MHD (between 15 and 30) with an MMD range between five and seven were also classified as CM patients.

CM-MOH was defined as patients with CM and AMD of triptans (and non-opioid analgesics, e.g. NSAIDs) for at least 10 days or non-opioid analgesics alone for at least 15 days per month (existing for at least 3 months) according to ICHD-3 criteria. [1]. CM patients who did not fulfil the MOH criteria were defined as CM-noMOH. Although there is no definition according to the ICHD-3 criteria, medication overuse in EM (EM-MO) was defined according to the criterion for MOH (AMD of triptans (and non-opioid analgesics) for at least 10 days or of non-opioid analgesics alone for at least 15 days per month (existing for at least 3 months)) but without fulfilling the criteria for CM (MHD < 15). EM patients with AMD of triptans (and non-opioid analgesics) for less than 10 days or of non-opioid analgesics only for less than 15 days were defined as EM without medication overuse (EM-noMO). 

All patients received either erenumab, fremanezumab or galcanezumab. No detoxification of acute medication was performed during or before the start of the CGRP (receptor) antibody therapy. Additionally, all patients received information about MO and the recommendation to reduce their acute medication in case of overuse. There was no prior withdrawal in our centre. We had no data on whether withdrawal had taken place in the past before treatment at our centre.

Data were collected for up to one year. To avoid attrition bias due to excluded data at the last time point (e.g., treatment discontinuation due to insufficient therapy), we analysed the difference between baseline value and the last observation time point (LOTP) within the first year of therapy for each patient. A paired Wilcoxon test was used to assess the effectiveness in reducing MHD, MMD and AMD from baseline (start of therapy) to the LOTP within the first year of therapy. Bonferroni correction for multiple testing was performed (significance level α = 0.017). We assessed statistically significant differences in 30% responder rates between patients with CM and MOH and patients with CM without MOH by Pearson's Chi-squared test (significance level α = 0.017). A null hypothesis significance test (NHST) and a minimal effect test via two one-sided tests (TOST) were performed with an alpha-level of α = 0.05. These tested the null hypotheses that true mean difference is equal to 0 (NHST), and true mean difference is greater than the equivalence ranges of -0.2 or less than 0.2 (TOST). To evaluate differences of treatment effectiveness between erenumab, fremanezumab and galcanezumab, responder rates were analysed using Kruskal–Wallis test. Analysis and visualisation were performed using R (version 4.3.2) and Office Professional Plus 2019 (Microsoft Corporation, Redmond, Washington, USA).

## Results

**Fig. 1:**
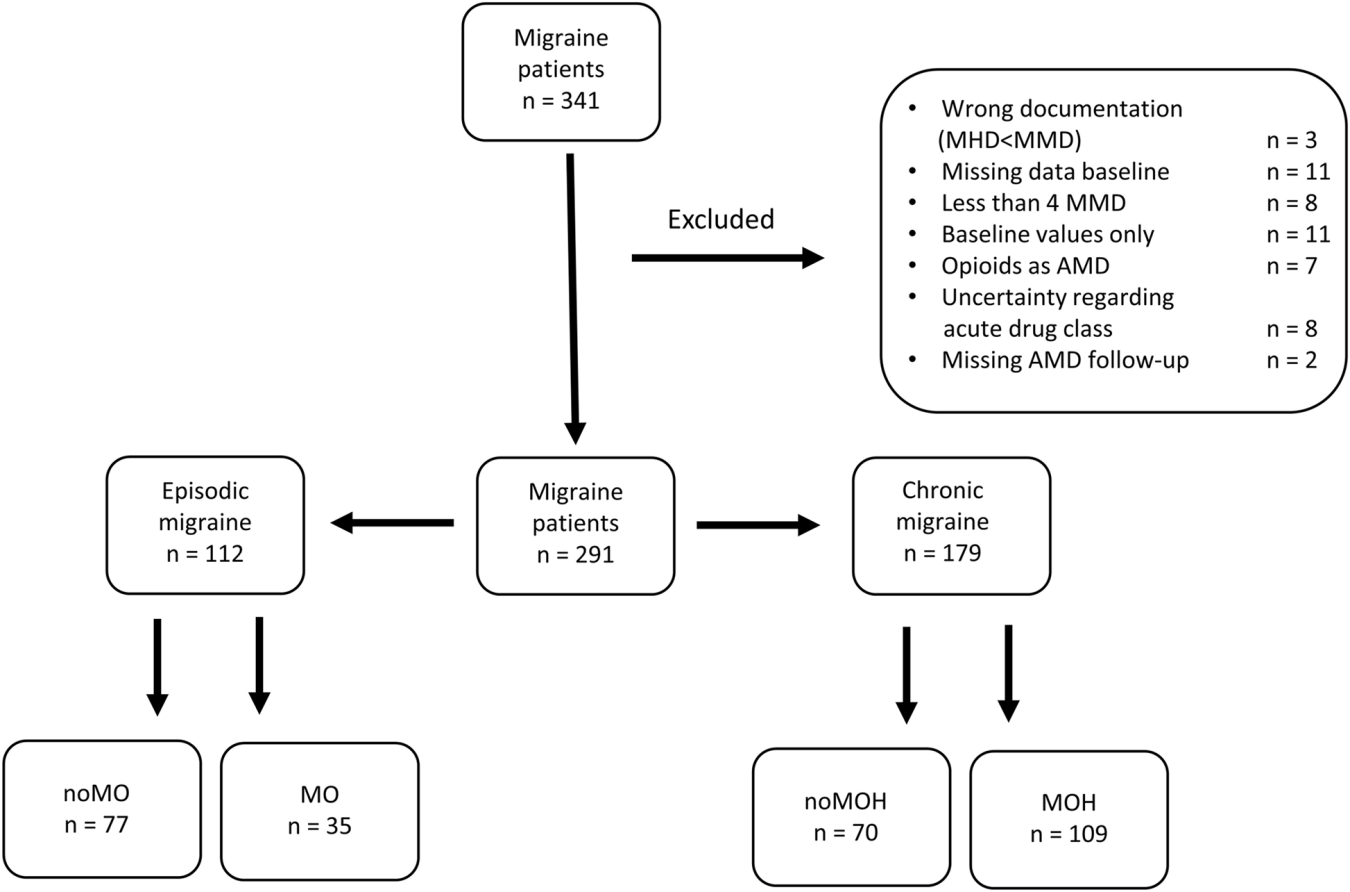
Patients included in study. (AMD: monthly acute drug intake, MHD: monthly headache days, MMD: monthly migraine days, MO: medication overuse, MOH: medication overuse headache)

In total, 341 patients were screened. After exclusion of 50 patients due to incomplete or implausible data, 291 patients were included in the analysis (Fig. 1). All patients were divided into either EM-MO and EM-noMO or into CM-MOH or CM-noMOH. Sixteen patients had more than 15 MHD (up to 30 MHD) but between five and seven MMD at the start of therapy. These patients were also assigned to the group of patients with CM. Patients´ characteristics, respective antibody therapy and pretreatment are shown in Table 1.

**Table 1:** Characteristics of migraine patients

	Episodic Migraine			Chronic Migraine		
	MO (*N* = 35)	No MO (*N* = 77)	Total (*N* = 112)	MOH (*N* = 109)	No MOH (*N* = 70)	Total (*N* = 179)
**Age**						
Mean (SD)	50.7 (9.1)	45.4 (12.0)	47.1 (11.4)	47.8 (10.8)	42.8 (13.5)	45.8 (12.1)
Range	25—66	23—68	23—68	20—77	19- 75	19—77
**Sex**						
Male	8 (22.9%)	6 (7.8%)	14 (12.5%)	20 (18.3%)	20 (28.6%)	40 (21.7%)
Female	27 (77.1%)	71 (92.2%)	98 (87.5%)	89 (81.7%)	50 (71.4%)	139 (77.7%)
Diverse	0 (0.0%)	0 (0.0%)	0 (0.0%)	0 (0.0%)	0 (0.0%)	0 (0.0%)
**CGRP antibody**						
Erenumab	19 (54.3%)	37 (48.1%)	56 (50.0%)	73 (67.0%)	44 (62.9%)	117 (65.4%)
Fremanezumab	10 (28.6%)	24 (31.2%)	34 (30.4%)	20 (18.3%)	16 (22.9%)	36 (20.1%)
Galcanezumab	6 (17.1%)	16 (20.8%)	22 (19.6%)	16 (14.7%)	10 (14.3%)	26 (14.5%)
**Aura**						
N-Miss	1	1	2	2	6	8
No	19 (55.9%)	54 (71.1%)	73 (66.4%)	56 (52.3%)	38 (59.4%)	94(55.0%)
Yes	15 (44.1%)	22 (28.9%)	37 (33.6%)	51 (47.7%)	26 (40.6%)	77 (45.0%)
**Premedication beta blocker**						
N-Miss	0	1	1	1	0	1
No	10 (28.6%)	17 (22.4%)	27 (24.3%)	24 (22.2%)	16 (22.9%)	40 (22.5%)
Yes	25 (71.4%)	59 (77.6%)	84 (75.7%)	84 (77.8%)	54 (77.1%)	138 (77.5%)
**Premedication topiramate**						
N-Miss	0	1	1	0	0	0
No	12 (34.3%)	25 (32.9%)	37 (33.3%)	22 (20.2%)	17 (24.3%)	39 (21.8%)
Yes	23 (65.7%)	51 (67.1%)	74 (66.7%)	87 (79.8%)	53 (75.7%)	140 (78.2%)
**Premedication flunarizine**						
N-Miss	0	1	1	2	0	2
No	19 (54.3%)	43 (54.4%)	62 (54.4%)	51 (47.7%)	35 (50.0%)	86 (48.6%)
Yes	16 (45.7%)	36 (45.6%)	52 (45.6%)	56 (52.3%)	35 (50.0%)	91 (51.4%)
**Premedication amitriptyline**						
N-Miss	0	1	1	1	0	1
No	10 (28.6%)	19 (25.0%)	29 (26.1%)	20 (18.5%)	13 (18.6%)	33 (18.5%)
Yes	25 (71.4%)	57 (75.0%)	82 (73.9%)	88 (81.5%)	57 (81.4%)	145 (81.5%)
**Premedication valproate**						
N-Miss	1	2	3	0	1	1
No	25 (73.5%)	58 (77.3%)	83 (76.1%)	72 (66.1%)	44 (63.8%)	116 (65.2%)
Yes	9 (26.5%)	17 (22.7%)	26 (23.9%)	37 (33.9%)	25 (36.2%)	62 (34.8%)
**Premedication Onabotulinumtoxin A**						
N-Miss	0	2	2	0	0	0
No	20 (57.1%)	42 (56.0%)	62 (56.4%)	23 (21.1%)	14 (20.0%)	37 (20.7%)
Yes	15 (42.9%)	33 (44.0%)	48 (43.6%)	86 (78.9%)	56 (80.0%)	142 (79.3%)

Patients in all four groups (EM-MO, EM-noMO, CM-MOH, CM-noMOH) showed a significant reduction in MHD, MMD and AMD at the LOTP from baseline to CGRP (receptor) antibody therapy (Table 2). Fifty-seven patients had their LOTP at 3 months, 51 patients at 6 months, 80 patients at 9 months and 103 patients at 12 months (mean follow up: 8.4 months).

**Table 2:** Treatment response at the LOTP

		MHD, Median (IQR)				MMD, Median (IQR)				AMD, Median (IQR)			
		Baseline	LOT	Reduction	*p*-value	Baseline	LOT	Reduction	*p*-value	Baseline	LOT	Reduction	*p*-value
**Episodic Migraine**	**MO**	12	5.7	-6	< 0.0001	11	4.3	-6.3	< 0.0001	11	5	-6.3	< 0.0001
		(10.2, 12.9)	(4.0, 8.2)	(-7.8, -3.0)		(10.0, 11.9)	(2.2, 7.0)	(-9.2, -3.8)		(10.0, 12.2)	(3.0, 6.8)	(-8.2, -4.2)	
	**noMO**	9.3	6.2	-3.3	< 0.0001	8	4.7	-3.3	< 0.0001	7	4	-1.7	0.0005
		(8.0, 11.7)	(3.7, 9.7)	(-5.3, -1.0)		(6.0, 9.3)	(3.0, 7.3)	(-5.0, -1.4)		(5.0, 8.0)	(2.7, 7.0)	(-4.3, 0.3)	
	**All**	10	6	-4.2	< 0.0001	8.7	4.7	-4	< 0.0001	8	4.3	-3.3	< 0.0001
		(8.3, 12.0)	(3.7, 9.3)	(-6.4, -1.3)		(6.7, 11.1)	(2.7, 7.3)	(-6.3, -1.7)		(6.0, 10.0)	(2.9, 7.1)	(-5.8,—0.6)	
**Chronic Migraine**	**MOH**	20.3	12.7	-6.7	< 0.0001	15	8.3	-5.4	< 0.0001	15	8.7	-6.3	< 0.0001
		(17.0, 26.0)	(7.3, 22.0)	(-13.3, -1.7)		(12.3, 20.0)	(5.7, 14.3)	(-11.3, -1.7)		(10.7, 19.0)	(5.0, 11.7)	(-9.3, -2.0)	
	**noMOH**	20.5	17.2	-3.3	< 0.0001	12.6	7.7	-3	< 0.0001	7.5	5.3	-2	< 0.0001
		(16.2, 30.0)	(10.1, 29.6)	(-9.2, 0.0)		(9.0, 16.5)	(5.0, 15.8)	(-6.0, 0.0)		(5.0, 9.0)	(3.1, 7.5)	(-3.7, 0.0)	
	**All**	20.3	14.7	-5.7	< 0.0001	15	8.3	-4.7	< 0.0001	10	6.3	-3.7	< 0.0001
		(16.7, 26.7)	(8.7, 24.8)	(-12.0, 0.0)		(11.0, 20.0)	(5.0, 14.7)	(-9.3, -1.0)		(8.3, 15.0)	(4.0, 10.3)	(-7.9, -1.0)	

*AMD* monthly acute drug intake, *EM-MO* Episodic migraine with medication overuse, *EM-noMO* Episodic migraine without medication overuse, *CM-MOH* chronic migraine with medication overuse headache, *CM-noMOH* chronic migraine without medication overuse headache, *IQR* interquartile range, *LOTP* last observation time point, *MHD* monthly headache days, *MMD* monthly migraine days, *MO* medication overuse, *MOH* medication overuse headache

**Fig. 2:**
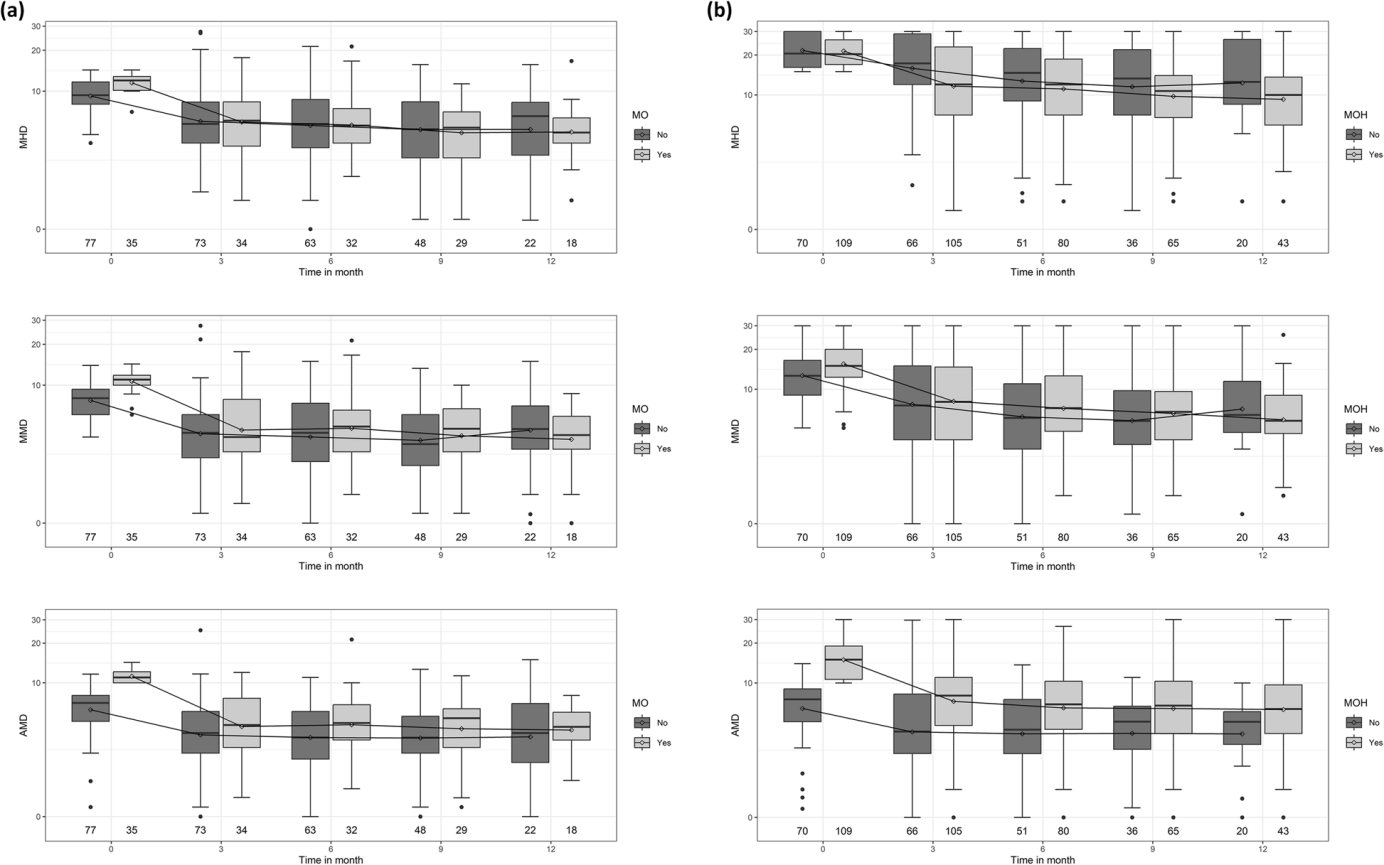
Treatment response up to 12 months. Development of MHD, MMD and AMD before and during treatment for EM with and without MO (**a**) and CM with and without MOH (**b**) up to 12 months. The number below shows the analysed patients at the respective time point. (AMD: monthly acute drug intake, MHD: monthly headache days, MMD: monthly migraine days, MO: medication overuse, MOH: medication overuse headache)

Changes in MHD, MMD and AMD with CGRP antibody therapy at each time point up to 12 months are shown in Fig. 2.

Table 3 shows the responder rates for CM patients with and without MOH and EM patients with and without MO.

**Table 3:** Responder rates at the LOTP

	Episodic Migraine	Chronic Migraine		
	Total (*n* = 112)	noMOH (*N* = 70)	MOH (*N* = 109)	Total (*N* = 179)
> = 30% reduction in MHD from baseline	68 (60.7%)	29 (41.1%)	61 (56.0%)	90 (50.3%)
> = 30% reduction in MMD from baseline	75 (67.0%)	33 (47.1%)	68 (62.4%)	101 (56.4%)
> = 30% reduction in AMD from baseline	69 (61.6%)	37 (52.9%)	72 (66.1%)	109 (60.9%)
> = 50% reduction in MHD from baseline	46 (41.1%)	16 (22.9%)	42 (38.5%)	58 (32.4%)
> = 50% reduction in MMD from baseline	54 (48.2%)	22 (31.4%)	45 (41.3%)	67 (37.4%)
> = 50% reduction in AMD from baseline	49 (43.8%)	23 (32.9%)	48 (44.0%)	71 (39.7%)

*AMD* monthly acute drug intake, *LOTP* last observation time point, *MHD* monthly headache days, *MMD* monthly migraine days

**Fig. 3:**
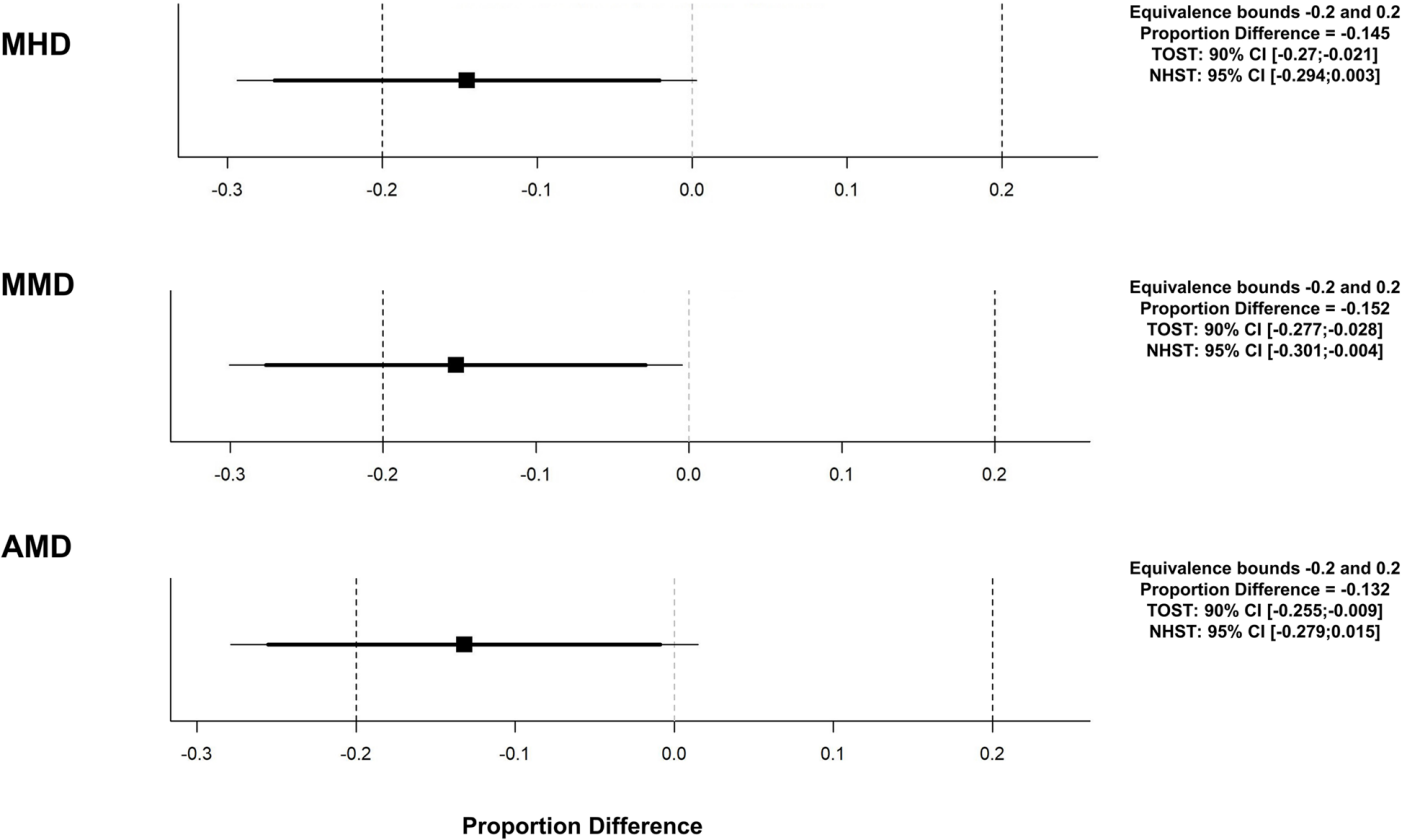
Equivalence analysis of 30% responder rates of CM patients with and without MOH. Equivalence bounds was set to 20%. Mean differences (black squares) and 90% confidence intervals (CIs; thick horizontal lines) and 95% CIs (thin horizontal lines) with equivalence limits ΔL = -.2 and ΔU = .2 showing whether the difference in responder rates between the two groups CM-noMOH and CM-MOH is statistically equivalent or not (TOST: two one-sided tests) and statistically different from zero or not (NHST: null hypothesis significance tests)

After up to one year of antibody therapy, MHD and AMD 30% responder rates were comparable for CM-MOH and CM-noMOH (MHD: CM-MOH: 56.0% vs. CM-noMOH: 41.1%, *p* = 0.058; AMD: CM-MOH: 66.1% vs. CM-noMOH: 52.9%, *p* = 0.077). Patients with MOH had a higher 30% responder rate for MMD compared to CM-noMOH patients (CM-MOH: 62.4% vs. CM-noMOH: 47.1%, *p* = 0.045) (Fig. 3). Nevertheless, it was not significant after Bonferroni correction (α = 0.017).

**Fig. 4:**
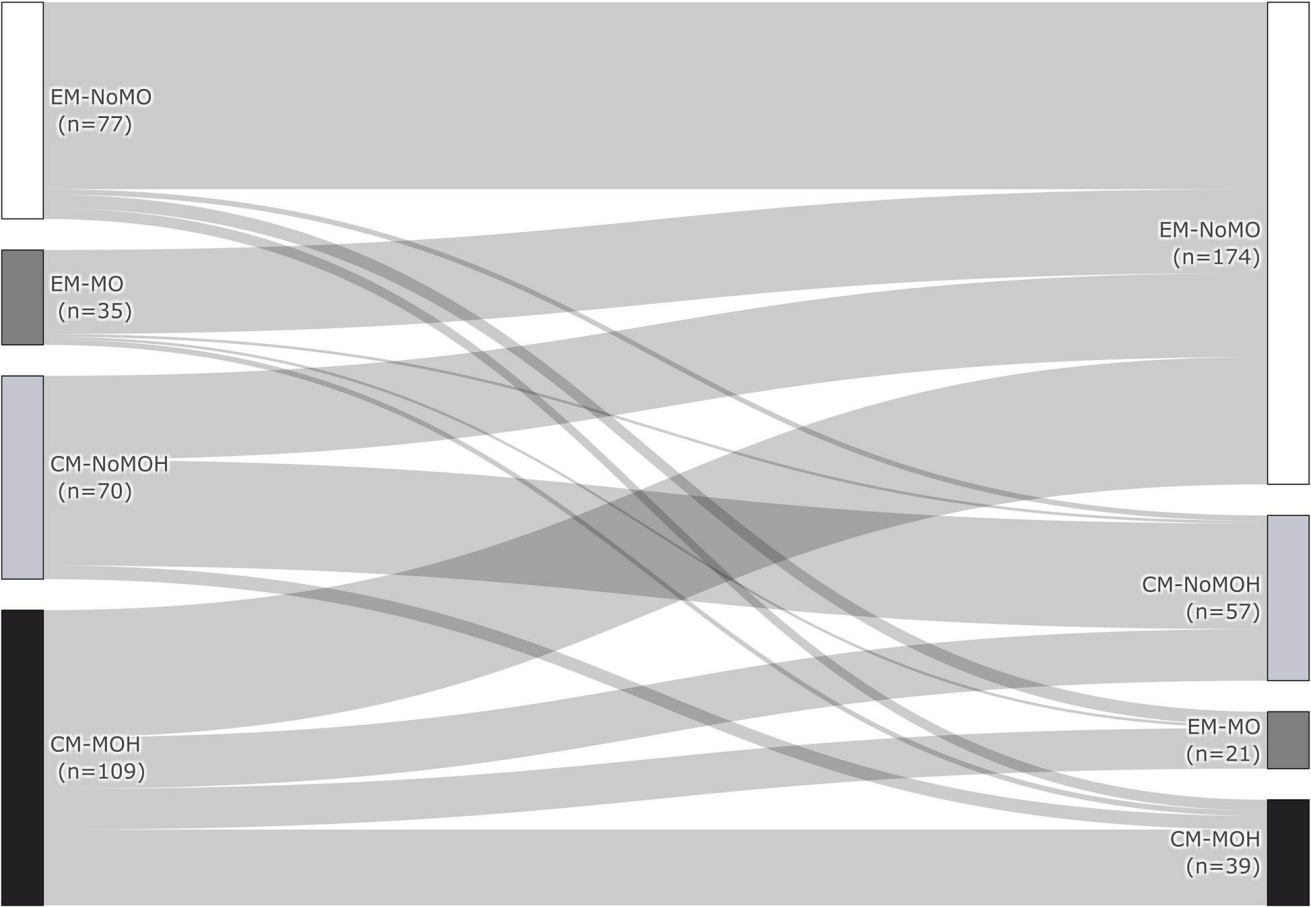
Changes of migraine type and MO/MOH during CGRP (receptor) antibody therapy. Migraine type and MO/MOH at baseline and at the LOTP (CM-MOH: chronic migraine with medication overuse headache, CM-noMOH: chronic migraine without medication overuse headache, EM-MO: Episodic migraine with medication overuse, EM-noMO: Episodic migraine without medication overuse, LOTP: last observation time point)

Regarding MO and MOH, changes of the respective group were analysed from baseline to the LOTP. In patients with EM and MO, 88.6% (31/35) lost their overuse during treatment, 44.3% (31/70) of CM-noMOH converted to EM-noMO. In patients with CM and MOH, 60.6% (66/109) no longer satisfied MO or MOH definition and 13.8% (15/109) had only EM-MO. However, a small number of patients showed no response or worsened under treatment in terms of MO and MOH (EM-noMO: worsened 14.3% (11/77); EM-MO: unchanged 2.9% (1/35), worsened 8.6% (3/35); CM-noMOH: unchanged 55.7% (39/70), worsened 7.1% (5/70); CM-MOH: unchanged 25.7% (28/109)). Changes are shown in Fig. 4.

**Fig. 5:**
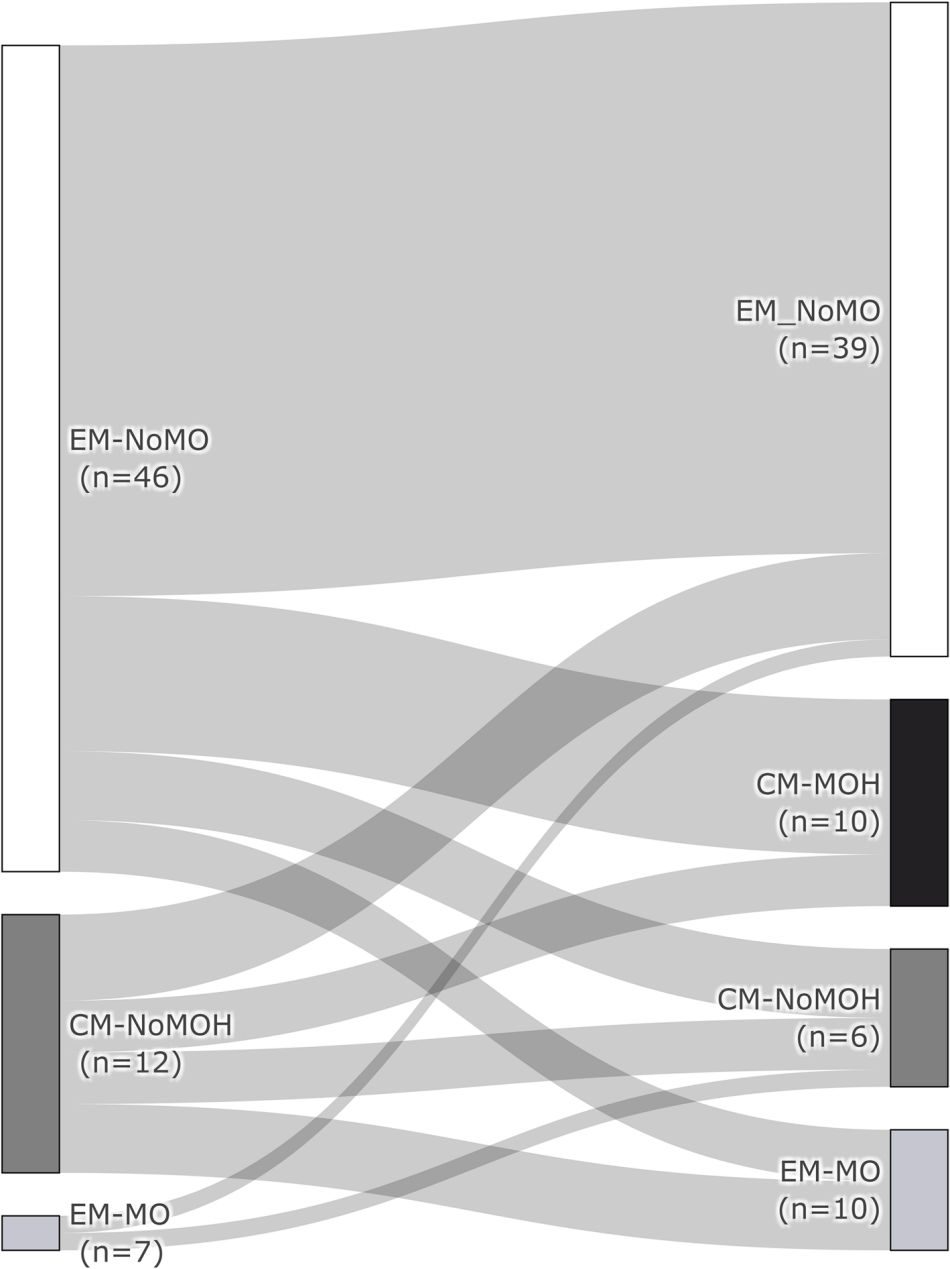
Changes of migraine type and MO/MOH after initial response to CGRP (receptor) antibody therapy. Migraine type and MO/MOH after three months of therapy and at the LOTP (CM-MOH: chronic migraine with medication overuse headache, CM-noMOH: chronic migraine without medication overuse headache, EM-MO: Episodic migraine with medication overuse, EM-noMO: Episodic migraine without medication overuse, LOTP: last observation time point)

The relapse rate was determined. Hundred-and-six of 109 CM-MOH patients had their first observed treatment time point at three months. In total, 22.6% (24/106) showed no response at this time point and still fulfilled the criteria for CM-MOH, 77.4% (82/106) improved and were classified as either CM-noMOH (14.6%; 12/82), EM-MO (8.5%; 7/82) or EM-noMO (56.1%; 46/82). Sixty-five patients who responded to the CGRP (receptor) antibody therapy had their LOTP beyond 3 months, the mean observation period was 10.3 months (LOTP: six months: *n* = 10, nine months *n* = 17, twelve months *n* = 38). Ten of 65 patients experienced a recurrence of CM-MOH (relapse rate: 15.4%). Eight patients (12.3%) worsened but without fulfilling the criteria for CM-MOH again. However, ten patients (15.4%) improved in terms of MO and MOH (Fig. 5).

No significant difference in reduction of MHD, MMD or AMD was observed in the CM-MOH group with respect to the different CGRP antibody therapies (Table 4).

**Table 4:** MHD, MMD and AMD reduction of CM-MOH patients at the LOTP depending on the respective CGRP (receptor) antibody

	Erenumab (*n* = 73)	Fremanezumab (*n* = 20)	Galcanezumab (*n* = 16)	Total (*n* = 109)	*p*-value
MHD Median (IQR)	-6.7 (-12.7, -2.5)	-4.0 (-15.7, 0.1)	-9.4 (-13.2, -2.0)	-6.7 (-13.3, -1.7)	0.694
MMD Median (IQR)	-5.3 (-10.0, -1.3)	-6.5 (-11.9, -1.7)	-7.8 (-12.8, -4.7)	-5.4 (-11.3, -1.7)	0.4
AMD Median (IQR)	-6.0 (-9.3, -1.7)	-7.7 (-9.5, -4.0)	-6.5 (-13.5, -2.8)	-6.3 (-9.3, -2.0)	0.234

*AMD* monthly acute drug intake, *LOTP* last observation time point, *MHD* monthly headache days, *MMD* monthly migraine days

## Discussion

Our study confirms the beneficial effect of CGRP (receptor) antibody therapy in treatment of migraine with MOH or MO under real-world conditions up to one year, even without any withdrawal of acute medication. Regarding MO of EM patients, 88.6% (31/35) lost their overuse during treatment. In CM patients, 74% (81/109) of CM-MOH patients improved under therapy. In detail, 19 patients (17%) lost their MOH but still had a CM, 47 patients (43%) converted to an EM without MO and 15 patients (14%) converted to an EM, but still had MO. However, 5% (9/182; all patients without MOH at baseline) worsened under CGRP (receptor) antibody therapy and developed a CM-MOH.

Regarding CM, there was no reduced response in the CM-MOH group compared to CM patients without MOH. In addition, we measured a tendency for even better treatment effect in terms of 30% responder rates for MMD in the MOH group compared to CM patients without MOH, but without statistically significance after Bonferroni correction. Moreover, only 15.4% had a relapse and fulfilled the CM-MOH criteria after a successful treatment initiation at the end of the observation (mean observation period: 10.3 months) (Fig. 4).

There was no significant difference in the reduction of MHD, MMD and AMD between patients who were treated with either erenumab, fremanezumab or galcanezumab. Therefore, the data does not indicate a preference for a specific antibody in the treatment of MOH.

According to ICHD-3 and unlike MOH, there is no official definition of MO in EM. A recent study defined acute medication overuse as AMD ≥ 10, but without distinguishing between triptans and other drugs [13]. Therefore, we used the criteria for MO used in the ICHD-3 criteria for MOH (distinguishing between non-opioid drugs only and triptans or drug combinations), but without fulfilling the definition of CM (< 15 MHD). This definition was already used elsewhere to describe MO in EM patients [14].

There is rising evidence regarding CGRP (receptor) antibody therapy in treatment of MOH, even without withdrawal of acute medication. In approval studies, subgroup analysis of erenumab, galcanezumab and fremanezumab showed a sufficient 50% MMD responder rate for patients with MO (erenumab 70/140mg: 36%/35%; galcanezumab 120/240mg: 27.1%/27.4%, fremanezumab quarterly/monthly: 34.8%/39.4%) (reviewed in [15]). These results are comparable to our data (50% MMD responder rate: CM-MOH: 41.3% vs. CM-noMOH 31.4%). An unblinded prospective real-world study on the treatment of CM patients with and without MOH demonstrated that the additional CGRP (receptor) antibody therapy was more effective than oral medication alone [16]. In a prospective study of 28 patients with inpatient withdrawal and 83 patients without inpatient withdrawal, participants were treated with either galcanezumab or erenumab. There were no differences in responder rates after three months of therapy (57% in the no-withdrawal group vs. 64% in the withdrawal group) [17]. However, the size of the groups differed significantly. Erenumab was effective after three months of treatment in a cohort of patients with CM and MOH under real-world conditions. A conversion to EM was observed in 64% of cases [18]. There was no control group of patients without MOH in this study.

Previous recommended drugs for the treatment of MOH were topiramate [19, 20] and onabotulinumtoxin A [21]. In a placebo controlled study, 50% MMD responder rate was 29% after 16 weeks and 22% at the end of the study (up to 23 weeks) for topiramate [19]. Nevertheless, there is conflicting evidence regarding the efficacy of topiramate for MOH treatment due to insufficient data. Also for onabotulinumtoxin A, data regarding responder rates for MOH patients are contradictory [8].

While the German guidelines recommend prophylactic therapy in combination with education (and withdrawal as a possible option) [22], international guidelines vary and are not consistent. Some guidelines recommend withdrawal of acute medication with simultaneous initiation of prophylactic therapy (reviewed in [23]). In a small randomised open-label study, patients with MOH were treated with either abrupt withdrawal (*n* = 20), prophylactic treatment without detoxification (*n* = 17) or no therapy (*n* = 19). After 12 months, there was no significant difference in the 50% responder rate for MHD (prophylactic treatment: 41% and withdrawal group: 25%, *p* = 0.081) [6]. An Italian real-world study analysed the effectiveness of erenumab and galcanezumab over 6 months in CM patients with and without MOH. It showed similar effectiveness in patients with CM alone and additional MOH (50% MMD responder rate: MOH: 63.6% vs. noMOH: 57.5%, *p* = 0.500). These responder rates were significantly higher than in our cohort. A possible reason may be that most of our patients were drug resistant to almost all previously approved therapies, indicating a therapy refractory cohort. In the Italian study, 60.6% (*n* = 60/99) were successful in stopping medication overuse [24]. This was similar to our data (Fig. 4).

A significant risk is relapse after successful treatment initiation. In 1996, a retrospective study showed a relapse rate of 25–38% after 120 days in MOH patients. Due to different diagnostic criteria and the overuse of various drugs (such as barbiturate-containing mixtures) [25], a comparison with our data is not possible. However, recent studies have also addressed this specific aspect of MOH treatment. A retrospective study analysed 124 MOH patients. Data were available up to 6 months for 102 patients (78 of whom had migraine). After withdrawal and optional concomitant prophylactic treatment, 39 patients (38%) had a relapse [26]. A prospective study analysed 96 MOH patients with various headache diseases after inpatient withdrawal. Data up to four years after withdrawal (*n* = 75) revealed a relapse rate of 32% for migraine patients [12]. Another prospective study of 240 patients with various primary headaches showed a relapse rate of 36.9% (*n* = 95) one year after withdrawal and during a prophylactic therapy (with amitriptyline, fluoxetine, propranolol, verapamil or divalproex sodium) [11]. In a retrospective Chinese study, 129 patients with MOH (migraine *n* = 97, tension-type headache *n* = 32) underwent a prophylactic treatment. After twelve months, the MOH relapse rate in the migraine group was 29.9% (*n* = 29) [9]. A prospective study analyzed 83 patients, treated with different therapy strategies (advice to reduce use, abrupt withdrawal or inpatient drug withdrawal program). In this study, the relapse rate for MOH after one year was 20.5% [10].

To our knowledge, relapse rate concerning MOH treatment with CGRP (reeptor) antibody therapy with or without acute medication withdrawal are not available to date. In our MOH group with successful treatment initiation, only 15.4% met the criteria for CM-MOH at the LOTP (mean observation period: 10.3 months). In contrast to other treatment options even without medication withdrawal, our data suggest a long-lasting effect of CGRP (receptor) antibodies in treatment and prevention of MOH.

Since we found no difference between MOH and noMOH, and if this is confirmed in further studies, drug withdrawal may in future be reserved for CGRP (receptor) antibody non-responders and patients with other overuse (e.g. opioids). As all patients were informed about the effects of MO, the effect of education alone cannot be assessed. However, since most patients were refractory to previous oral therapies (+ onabotulinumtoxin A in the case of CM) and were also informed about the consequences of an overuse as standard in previous treatments, the measured treatment effect by education alone is unlikely.

Limitations of this study are that all clinical routine data such as headache diaries, patient questionnaires and physician reports were collected retrospectively. In addition, the decrease in the number of patients at the end of the 12 months may include the error of having only well responding patients in the final cohort. To address this bias, the responder rates were analysed for the last documented value within 12 months. Drop-outs, changes in therapy and patients who continued the therapy were all included to reduce this bias. Another limitation is the small sample size, especially due to the subgroup analysis. Furthermore, the difference between MHD and MMD is difficult to determine retrospectively and is often erroneous, limiting the ability to interpret the difference. We also do not have sufficient data on other causes that may only lead to short-term analgesics overuse (e.g. fever, infection, injuries) that may mimic a response to treatment. A MO for other reasons, such as anticipatory anxiety, other chronic pain conditions or other additional types of headache could also not be sufficiently distinguished in our data, which could mimic non-response, at least in AMD. Finally, this is a single centre study, therefore it is currently not possible to universalise the results.

## Conclusion

This study supports the recommendation that CGRP (receptor) antibodies can be used for prophylactic therapy in patients with migraine and MO or MOH. Effectiveness seems not be reduced by MOH, our data shows even a tendency towards a better effect in patients with MOH. Since our data also demonstrates good effectiveness on MO in EM patients, a preventive effect on the development of MOH could also be assumed. In contrast to previous therapies regimes, the low relapse rate up to 12 months of treatment suggests a sustained effect of CGRP (receptor) antibodies in the treatment of MOH patients. If future controlled and randomised trials confirm the long-term effects of CGRP (receptor) antibody therapy and a low relapse rate also beyond one year, outpatient or inpatient withdrawal could be reserved for CGRP (receptor) antibody non-responders.

## Data Availability

The datasets used and/or analysed during the current study are available from the corresponding author on reasonable request.

## Abbreviations

AMD: Monthly acute drug intake
CGRP: Calcitonin gene-related peptide
CM: Chronic migraine
CM-MOH: Chronic migraine with medication overuse headache
CM-noMOH: Chronic migraine without medication overuse headache
EM: Episodic migraine
EM-MO: Episodic migraine with medication overuse
EM-noMO: Episodic migraine without medication overuse
IQR: Interquartile range
LOTP: Last observation time point
MHD: Monthly headache days
MMD: Monthly migraine days
MO: Medication overuse
MOH: Medication overuse headache
